# Supplementary material for: Digital mental health and peer support: Building a Theory of Change informed by stakeholders’ perspectives
Source: PLOS Digit Health. 2024 May 30;3(5):e0000522. doi: 10.1371/journal.pdig.0000522 (PMC11139267; doi:10.1371/journal.pdig.0000522)
Supplement: S1 Checklist — (DOC) [file pdig.0000522.s001.doc]

**COREQ (Consolidated Criteria for Reporting Qualitative Research)**

**32-item checklist**

**Manuscript:** Digital mental health and peer support: Building a Theory of Change informed by stakeholders’ perspectives.

**Developed from:** Tong A, Sainsbury P, Craig J. Consolidated criteria for reporting qualitative research (COREQ): a 32-item checklist for interviews and focus groups. *International Journal for Quality in Health Care*. 2007. Volume 19, Number 6: pp. 349 – 357

| **No. Item** | **Guide questions/description** | **Reported on Page #** |
| --- | --- | --- |
| **Domain 1: Research team and reﬂexivity** | | |
| *Personal Characteristics* | | |
| 1. Interviewer/facilitator | Which author/s conducted the interview or focus group? | P 10 – MT, GH, AM |
| 2. Credentials | What were the researcher’s credentials (e.g., PhD, MD)? | P10 |
| 3. Occupation | What was their occupation at the time of the study? | P10 |
| 4. Gender | Was the researcher male or female? | Not explicitly reported |
| 5. Experience and training | What experience or training did the researcher have? | P10 |
| *Relationship with participants* | | |
| 6. Relationship established | Was a relationship established prior to study commencement? | P6-9 this was variable as some participants only took part in interview/focus groups while others took part in both stakeholder events |
| 7. Participant knowledge of the interviewer | What did the participants know about the researcher (e.g., personal goals, reasons for doing the research)? | Not explicitly reported – participants knew the goals of the research and the researchers were employed to address these |
| 8. Interviewer characteristics | What characteristics were reported about the interviewer/facilitator (e.g., bias, assumptions, reasons and interests in the research topic)? | P10 |
| **Domain 2: study design** | | |
| *Theoretical framework* | | |
| 9. Methodological orientation and Theory | What methodological orientation was stated to underpin the study (e.g., grounded theory, discourse analysis, ethnography, phenomenology, content analysis)? | P4/5 and P9/10 |
| *Participant selection* | | |
| 10. Sampling | How were participants selected (e.g., purposive, convenience, consecutive, snowball)? | P6/7 |
| 11. Method of approach | How were participants approached (e.g., face-to-face, telephone, mail, email)? | P6/7 |
| 12. Sample size | How many participants were in the study? | P7 |
| 13. Non-participation | How many people refused to participate or dropped out? What were their reasons? | NA – participants opted in. No drop outs. |
| *Setting* | | |
| 14. Setting of data collection | Where was the data collected (e.g., home, clinic, workplace)? | P8 |
| 15. Presence of non-participants | Was anyone else present besides the participants and researchers? | NA |
| 16. Description of sample | What are the important characteristics of the sample (e.g., demographic data, date)? | P7 |
| *Data collection* | | |
| 17. Interview guide | Were questions, prompts, guides provided by the authors? Was it pilot tested? | P10 |
| 18. Repeat interviews | Were repeat interviews carried out? If yes, how many? | P8/9 |
| 19. Audio/visual recording | Did the research use audio or visual recording to collect the data? | P8 |
| 20. Field notes | Were ﬁeld notes made during and/or after the interview or focus group? | NA – everything recorded |
| 21. Duration | What was the duration of the interviews or focus group? | P8 |
| 22. Data saturation | Was data saturation discussed? | NA – data was collected from participants to identify key inputs, processes etc of the ToC and this was later reviewed by participants. We identified gaps and tried to address these but note the limitations of this in the discussion. |
| 23. Transcripts returned | Were transcripts returned to participants for comment and/or correction? | No – the resultant ToC was reviewed and commented on at the final stakeholder event |
| **Domain 3: analysis and ﬁndings** | | |
| *Data analysis* | | |
| 24. Number of data coders | How many data coders coded the data? | P9 |
| 25. Description of the coding tree | Did authors provide a description of the coding tree? | P9 |
| 26. Derivation of themes | Were themes identiﬁed in advance or derived from the data? | P10 |
| 27. Software | What software, if applicable, was used to manage the data? | P9 |
| 28. Participant checking | Did participants provide feedback on the ﬁndings? | P8/9 – final stakeholder workshop |
| *Reporting* | | |
| 29. Quotations presented | Were participant quotations presented to illustrate the themes/ﬁndings? Was each quotation identiﬁed (e.g., participant number)? | P11-17 |
| 30. Data and ﬁndings consistent | Was there consistency between the data presented and the ﬁndings? | P11-17 |
| 31. Clarity of major themes | Were major themes clearly presented in the ﬁndings? | P11-17 |
| 32. Clarity of minor themes | Is there a description of diverse cases or discussion of minor themes? | P11-17 |
