# Supplementary material for: Digital mental health and peer support: Building a Theory of Change informed by stakeholders’ perspectives
Source: PLOS Digit Health. 2024 May 30;3(5):e0000522. doi: 10.1371/journal.pdig.0000522 (PMC11139267; doi:10.1371/journal.pdig.0000522)
Supplement: Attachment — Submitted filename: Response to reviewers.docx [file pdig.0000522.s002.docx]

Dear editor and reviewers,

Thank you for your comments and suggestions. We feel following these the manuscript is much improved. All changes have been tracked and a list of responses/changes is set out below.

Editor: Thank you for the guidance for the journal requirements. I hope I have updated the relevant sections as required. I have updated the competing interests statement and financial disclosure statements. The figure of the ToC had already been added as a separate file – I have removed it from the main manuscript. I have also attached the COREQ checklist as a supplementary file.

Reviewer 1: Thank you for your comments. Yes we used the COREQ guidance which offers guidance on how to report qualitative data in manuscripts (I have added this as a supplementary file). It did not determine how data was collected, analysed or interpreted. We did use a pragmatic approach to understand how the Togetherall platform was understood in terms of inputs, use, outcomes, and contextual factors to develop a theory of change as perceived by the mentioned stakeholders. We did not use grounded theory as the theory of change describes what is occurring but does not develop a clear behaviour change theory – rather is considering all the influential factors which were identified using a thematic framework approach as described in the text. I checked the links to the data analysis references and all worked a part from one which I have replaced (number 40)

Reviewer 2: Thank you for your comments. I have updated Table 2. In terms of improving the strengths section I have added the following text: “The ToC can be used to understand areas for improvement for digital platforms themselves (i.e. focusing on engagement and integration) and to identify gaps for further research (i.e. understanding more clearly member experiences during use). The stakeholders in this research do suggest these platforms can be successfully incorporated into mental health support systems and the benefits this can offer for services (e.g. alleviating demand) and members (e.g. improving mental health awareness and understanding). Notably, the research does reveal key contextual influences such as digital poverty which could be used to inform decisions at a policy level”. We are aware of the limitations of the work given the difficulty in recruiting members for interview so are being mindful of this in our interpretations and details of the strengths and limitations.

Reviewer 3: Thank you for your comments. I have updated the introduction as per your suggestions by including the following:

More information on the theory of change and development:”By understanding these factors, public health interventions can be adapted and improved to be more effective in their implementation and results (32). As such a ToC enables identification of gaps in support, challenges in implementation, what if influencing the desired behaviour change and what factors may be influencing this (33). It can therefore be used as a framework for the intervention itself or as a plan on how to effectively deliver an intervention (35,36). For example, Lund and colleagues used the ToC approach to identify challenges in mental health support in low-and-middle income countries to support the development of a public health intervention. The ToC helped to identify key challenges including limited resources for mental health support, and high staff turnover (i.e. as a result the time to train new staff could inhibit access to support).”

Updating the rationale and reason for using the Togetherall platform. We have updated the togetherall platform section as follows: “Togetherall was selected as a representative of an online digital mental health platform combined with with moderated peer support due to its increasing use in the UK (29). It has been incorporated into a range of education, healthcare and employment settings therefore provided an opportunity to gain insights from a range of stakeholders.” We hope this alongside the updated ToC session strengthens the rationale more soundly.

The participant section has been moved to methods.

I have updated the discussion section to consider digital literacy and inclusion more fully: “Furthermore our study highlighted wider contextual factors as inhibiting engagement. This was in the form of digital literacy, inclusion and poverty. Stakeholders reasoned a degree of digital literacy was key as an initial input to the platform. Members who had a higher level of understanding of how to use the platform would continue to engage but those who experienced difficulty in accessing or navigating the website would disengage. Pertinantly, stakeholders reported internet provision (i.e. access to wifi or data or having an internet compatible device) was a barrier to uptake of the platform. Particularly in members who could not afford their own data and devices due to competing monetary commitments (e.g. energy bills).” And in the future research section “Similarly platforms and researchers need to consider how to make these resources more accessible to those who struggle with digital literacy or are experiencing digital poverty.”

Yours Sincerely,

Meigan Thomson
